# Is allergic sensitization relevant in severe asthma? Which allergens may be culprit?

**DOI:** 10.1186/s40413-016-0138-8

**Published:** 2017-01-06

**Authors:** Carlo Lombardi, Eleonora Savi, Erminia Ridolo, Giovanni Passalacqua, Giorgio Walter Canonica

**Affiliations:** 1 Departmental Unit of Allergology- Clinical Immunology, and Respiratory Diseases, Fondazione Poliambulanza, Via Bissolati, 57, Brescia, Italy; 2 Departmental Unit of Allergology, AUSL of Piacenza, “Guglielmo da Saliceto” Hospital, Piacenza, Italy; 3 Department of Clinical and Experimental Medicine, University of Parma, Parma, Italy; 4 Allergy and Respiratory Diseases, IRCCS San Martino-IST-University of Genoa, Genoa, Italy

**Keywords:** Severe asthma, Atopy, Allergic sensitization, Allergens

## Abstract

Severe asthma is a major health concern. The allergic (IgE-mediated) form of asthma is well known from a pathogenic viewpoint. We searched the available literature to identify which allergens are most frequently associated with severe, refractory or life threatening asthma. According to the results, molds, pet dander, cockroach and ragweed were more frequently responsible for severe asthma. Thunderstorm asthma, in addition, represents a special association between allergic sensitization and an external climatic factor. A detailed knowledge of the most harmful allergens is mandatory for an appropriate diagnostic and preventive approach.

## Background

Bronchial asthma, which prevalence is around 5-10% worldwide [1, 2], remains a major health problem in all age groups for patients, their families and the community. The asthma-related respiratory symptoms cause a limitation of everyday activity with consequent absenteeism/presenteism. Exacerbations may require extra visits, emergency room admissions, and hospitalizations. Of note, fatalities continue to be reported [3]. Indeed, in the majority of patients, asthma can be adequately treated with the standard of care therapy [4], and most patients achieve a satisfactory control of the disease. Nonetheless, a not negligible subgroup of subjects remains “difficult-to-treat” or “uncontrolled” despite adequate therapy. “Severe” or “difficult to treat” or “refractory” asthma is a heterogeneous condition that encompasses different phenotypes/endotypes, such as eosinophilic, obesity-related, neutrophilic, late onset asthma, and remains a major unmet need [5, 6]. Severe asthma accounts for only 5–10% of all cases, but it is responsible for the majority of direct and indirect costs. Thus, severe asthma poses a significant health care burden accounting for up to 50% of the asthma budget in developed and developing Countries.

Several multicenter cohort studies of patients with severe asthma have been published [7–10]. These studies shed light upon some of the emerging clinical characteristics of this challenging group of patients and provide important insights into the strategic priorities for its management. Managing patients with severe asthma is complex, and requires a multidisciplinary approach and a standardized protocol, in addition to a uniform definition. Asthma is a rare cause of mortality, contributing to less than 1% of all deaths in most countries worldwide. Rates of death rise almost exponentially from mid-childhood to older ages, so the majority of asthma deaths occur after middle age [11]. Although asthma mortality trends declined in many high-income countries, studies have suggested that avoidable factors still play a major role in preventing severe asthma or asthma deaths. There is increasing evidence that severe asthma phenotypes are related to genetic factors, age of onset, disease duration, exacerbations, rhinosinusal disease and inflammatory characteristics [12–15]. For instance, in a study in a general population, as part of the European Community Respiratory Health Survey (ECRHS), there was no gender difference in asthma severity in the two surveys. However, those studies suggested that asthma severity might be less stable in women than in men [16]. Occupational exposures have also been associated with late-onset, and more severe asthma [17].

The evidence for a favorable clinical response to environmental control in severe asthma remains so far inconsistent. Atopy and allergy have long been associated with asthma and, to some degree, with severe asthma. (Table 1) [7–10]. It is known that early-life exposures and sensitization to various allergens, occur in children with severe asthma [18], and the association between allergy and asthma severity is stronger in children [19, 20]. In general, the association among specific IgE sensitization (skin prick test or serum IgE assay), exposure and symptoms usually help to identify the factors contributing to the severity of disease [21]. Some specific allergens, in particular cockroach [22] and *Alternaria* [23] have been associated with more severe forms of asthma, and asthma deaths have been associated with high fungal spore counts in the environment [24]. In parallel, sensitization to mold has been associated with increased asthma severity and intensive care admissions in adults [25]. Another relevant aspect of severe asthma with allergic sensitization is the relationship with thunderstorms (“thunderstorm asthma”). An increasing body of evidence confirmed the occurrence of asthma epidemics during thunderstorms in pollen seasons, in various geographical areas [26]. Patients without asthma symptoms, but affected by seasonal rhinitis can also experience severe asthma attacks, and there is a link between thunderstorm asthma and pollen sensitizations [27, 28]. Identifying the possibly related causal allergens is mandatory for an appropriate therapeutic strategy [29, 30], although the evidence supporting secondary prevention of asthma (allergen avoidance) is still weak [31, 32].

**Table 1:** Demography and atopic status data from three of most representative studies on severe asthma

	Enfumosa study	Tenor study	SARP Study
Study design/location	Cross-sectional European multicentre study (9 Countries)	U.S. Multicentre Observational study	Observational cross-sectional multicentre study (9 sites in U.S. and 1 site in U.K.)
Patients (N.) with severe asthma	163	2282 (48% OF TOTAL)	204
Age characteristics	Age range: 17-65 yrs	Mean age ± SD : 38.9 ± 20.92 yrs	Mean age ± SD: 41 ± 13 yrs
Sex	Sex ratio (F/M) : 4.4 / 1	Not reported^c^	Females: 64%
≥1 Positive skin prick test	58% (VS. 78% OF Controlled asthmatics)	-	71% (VS. 85% of mild and 87% of moderate asthma)
Mean total serum IgE	109 KU/L-^1^, 95% CI: 85-139	106.6 IU/ml^a^	2.0 ± 7.6^b^
References	2	3	4

^a^Overall, the TENOR study patients with severe asthma had elevated geometric mean IgE values compared with patients with moderate and mild asthma. IgE values for children and adolescents increased with asthma severity

^b^Total serum IgE (log)

^c^Overall patients (mild/moderate/severe asthma): females : 2945 (62.2%); males : 1792 (37.8%)

To assess the impact of allergenic sensitization on severe asthma, a search of current scientific literature was performed and the results were filtered to identify relevant articles or studies with data from well-designed clinical studies in human patients. A PubMed literature search was performed using the Boolean string severe asthma* [title/abstract] AND allergic sensitization [title/abstract] AND atopy [ title/abstract].

## Mold sensitization and severe asthma

Allergy is one of the major pathophysiological aspects of asthma requiring hospitalisation, and the concentration of IgE antibodies is relevant in severe allergic asthma, as happens for instance in exacerbations due to rhinovirus infections [33]. In principle, any allergen can cause severe asthma in sensitized patients. Among children admitted to hospital for acute exacerbations, more than 85% are allergic. Of note, allergen-carrying particles with a diameter ≤ 5 microns such as HDM, dog, cat dander and fungal spores (*Aspergillus, Penicillium, Alternaria)* can entry the lower airways, thus more likely to cause asthma. In this context, it is important to distinguish between sensitization to thermotolerant fungi which can colonise the airways (mainly Aspergillus, Penicillium and Candida) so providing a persistent allergenic stimulus in the absence of airborne exposure and non-thermotolerant allergens such as Cladosporium and Alternaria where allergenic effects are directly related to airborne content of the spores. The former are associated with lung damage, whereas the latter are more closely associated with epidemic thunderstorm episodes. Several studies have found that there is a correlation between the severity of allergic diseases and the proteolytic activity of *Alternaria* extracts [34]. Furthermore, a possible key role of sensitization to *Alternaria* species has been observed in thunderstorm related severe asthma [35, 36]. In the specific case *Alternaria*, the prerequisites for severe asthma epidemics associated with thunderstorms are: sensitization, current asthma, sudden large allergen exposure, thunderstorm with cold outflow occurring at a time and location during an allergen season, in which many asthmatics are outdoors, sudden release of large amounts of respirable allergenic fragments, particularly fungal spores such as *Alternaria* [35–38]. Mold sensitivity is usually associated with increased severity of asthma and hospital admission in adults, while in children is related to increased bronchial hyperactivity [39]. Asthma-related deaths are more frequent in young adults during spore peaking [26, 40]. Molds are an important causative agent of severe asthma as thus the term SAFS (Severe Asthma associated with Fugal Sensitization) has been coined to identify those patients with severe asthma, not-responsive to standard therapy [41]. The exact prevalence of mold sensitivity is not well defined, but it is believed to range between 13% and 78% in atopic patients [42, 43]. Several species of fungi are considered responsible for allergic sensitization, but the more relevant belong to the Ascomycetes family: *Alternaria, Aspergillus, Botrytis, Epicoccum, Fusarium* and *Penicillium* species [41]. Sensitization to *Alternaria* and *Cladosporium* species are more frequently associated with persistent and severe asthma [41].

Molds are both outdoor and indoor persistent allergens, although with seasonal variations, that depend on climatic factors (rain, humidity, temperature, air pollutants and wind). Indoor spores can derive from molds present outdoor, or from inside damp and moist walls in buildings. Rain is usually necessary for spores discharge in air, not also from outdoor, but even from house surfaces, especially during thunderstorms [44]. Each species of fungi presents several allergens, that can be structural components of the cell and/or derived from metabolic products [45]. Currently 30 allergens from *Aspergillus sp*, 16 from *Penicillium sp*, 10 from *Cladosporium sp* and 9 from *Alternaria* sp have been identified. Although there is some documented cross-reactivity among allergens [46, 47], also species-specific allergens, such as Asp 1 and Alt 1, have been characterized [46, 47]. Exposure to fungal allergens can cause severe asthma trough 3 different mechanisms: a) inhalation of spores or hyphae, that act as allergens in patients sensitized; b) fungal colonization of airway, i.e. allergic bronchopulmonary aspergillosis (ABPA), associated to sensitization to fungal antigens; c) fungal colonization of districts different from respiratory tract (generally skin) in patients sensitized to involved fungi [33]. This wide spectrum of clinical entities in patients with sensitization to fungi is due to their potent antigenicity and immunomodulatory activities [48]. Immunopathogenesis is mediated by β-glucans and theirs receptor, dectin-1. In fact, after the recognition of β-glucans, dectin 1 on macrophages surface triggers the release and the production of several inflammatory mediators [49]. Recent finding suggest that the immune response to fungi directs T cell towards Th17 cells, through dendritic cells action. In the same studies the Authors demonstrated that induction of Th17 cells strictly correlates with severe allergic asthma, with possible steroid resistance [46]. Fungal proteases act as allergens, and also as mediators of tissue injury, inducing production of IL-6 and IL-8, proinflammatory citokines involved in exacerbations of asthma. Moreover, it has been demonstrated that the receptor activated by protease (PAR) type 2 is overexpressed in bronchial tissue from asthmatic patients, suggesting a potential increased vulnerability to fungi in these subjects [42]. In case of colonization of the airways a Th-2 hypersensitivity mechanism is involved. Disease is characterized by increased severity of asthma, increased IgE serum level, transient infiltrates in lungs, presence of IgG and IgE against molds [33, 42]. The most common fungi implicated in pulmonary colonization are from *Aspergillum* species, less frequently *Candida*, *Curvularia* and *Penicillium* species can be involved [50]. Asthma therapy in this case is associated with antifungal systemic treatment [42]. The third mechanism was firstly proposed in 1930 by Wise et al., who observed that patients with skin and/or nails fungal infection presented an increased asthma severity [51], and a significant correlation with sinusitis, rhinitis and urticaria associated to fungal colonization of skin. Supporting evidences to this hypothesis are derived from several trials in which patients affected by severe asthma and fungal skin infection had a relevant improvement in asthma control after itraconozole and fluconazole [52, 53]. Sensitization to Asp f 1 and/or Asp f 3 may be more indicative of allergic asthma. [54]. It was also observed that pediatric severe asthma with fungal sensitization was associated with more oral steroid therapy and higher IL-33 levels [55].

## Pollen sensitization and severe asthma

The relationship occurring between pollen sensitisation and asthma severity has been widely studied, and an abundant literature on this aspect is currently available [13, 33]. The IgE antibody profile for a broad spectrum of allergen molecules, exhaled nitric oxide (FEno) and bronchial responsiveness were assessed in asthma patients. Asthmatic patients showed more frequently sensitization to grass, tree, weed pollens, furry animals, mold, latex and foods of plant origin. Asthma prevalence was increased in patients with food-pollen-perennial sensitisation. In this group of patients also FEno and bronchial responsiveness were increased with respect to groups of patients with sensitization only to pollen , or food or perennial [56]. Some evidence suggested that air pollution may interact with airborne allergens enhancing the risk of atopic sensitization and exacerbation of symptoms in sensitized subjects. These phenomena are supported by current in vitro and animal studies showing that the combined exposure to air pollutants and allergens may have a synergistic or additive effect on asthma and allergies [57]. Ragweed pollen sensitization induces asthma much more frequently than other pollens [58]. Concerning *Olea* sensitization, the association between the presence of asthma and sensitization to Ole e 7 resulted statistically significant [59]. To date, among pollens, only grass, *Parietaria (*Wall Pellitory) and *Olea* pollen have been suggested as possible triggers in thunderstorm-related asthma [60]. A thunderstorm-related asthma episode was observed in Naples (Italy) on June 2004, when six adults and one child received emergency treatment. All patients showed allergic respiratory symptoms upon exposure to *Parietaria* pollen but they were not sensitized to grasses [61]. Losappio et al. observed 20 patients with allergic sensitization to Olea pollen brought to an emergency department in Barletta, Italy, for sudden and severe asthmatic symptoms in May 2010 following a violent thunderstorm [36]. On the basis of these observations, all subjects affected by pollen allergy should be alerted to the danger of being outdoors during a thunderstorm in the pollen season, as such events may be an important cause of severe asthma exacerbations.

## Animal dander sensitization and severe asthma

Pet allergy is considered one the most important causes of severe or uncontrolled asthma. A specific IgE response to more than three animal-derived components was observed to be more common among uncontrolled severe asthmatics children compared to those with controlled asthma [62]. Component Resolved Diagnosis (CRD) allows the identification of specific allergens associated with the severity of asthma and the identification of cross reactive molecules that are clinically significant as well as Can f 6 or Fel d 4. They are lipocalin allergens and could explain the role in cross reactivity of dog with cat and horse. The physician can advise on whether or not to keep a household pet or which species could be tolerated. This may have a huge impact on the child’s well-being [63]. CRD allows to evaluate the pattern of sensitization to pet IgE components and its association with clinical symptoms and their severity. Specific IgE to Can f 2 was significantly associated with asthma diagnosis, Can f 3 with moderate/severe rhinitis and asthma diagnosis, Can f 5 with persistent and moderate-severe rhinitis, and Equ c 3 with persistent rhinitis and severe asthma [64]. Furthermore, the sensitization to Can f 2 and Equ c 1 was more common in severe asthma than in children with controlled asthma [65]. Asthmatic children with cat allergy have higher Fel d 1-specific IgE levels as compared to those with rhinitis alone, and this suggests that high IgE levels to Fel d 1 could be a marker of increased asthma risk [66]. Fel d 2 is a serum albumin, abundant in saliva and dander. It is a highly cross-reactive molecule, associated with cat – pork syndrome but only a small number (10-20%) of patients sensitised to Fel d 2 report immediate reactions to beef, whereas sensitization to Fel d 2 is associated with more severe respiratory symptoms [67].

## Cockroach allergens sensitization and severe asthma

Cockroach allergy is recognized as an important cause of asthma and cockroach-induced asthma was described as a more severe disease, associated with perennial symptoms and high levels of total IgE. Cockroaches produce several allergens that induce sensitization, and exposure to high levels of cockroach allergens at home is a major risk factor for symptoms in sensitized individuals [68]. Recent data suggest that cockroach allergen may be a most relevant urban allergen exposure and may be related to asthma severity [69]. Indoor allergen exposure in inner-city areas has been of particular interest given that patients living in urban areas have increased asthma severity, decreased asthma control, and greater health care use [70]. Rosenstreich et al. found that cockroach allergens are easily detectable in inner-city homes [22]. Furthermore, this study demonstrated that asthmatic children sensitized and exposed to high levels of cockroach allergens had an increased asthma morbidity. Ahluwalia et al. observed that in an inner-city community with high exposure to cockroach allergens, there was a more strongly and consistent association with poor asthma outcomes [71]. Gelber et al. and Call et al. showed that cockroach sensitization was more common in asthmatics compared to non-asthmatics referred to an emergency room. When sensitization was associated with direct exposure, a very high association with asthma occurred. [72, 73]. In a study carried out in Thailand, the possible changes in disease severity and allergen sensitization of children with asthma in an interim period of 5 years was evaluated [74]. During the years 2004-2009, asthma severity increased with increasing sensitization to mite and mite plus cockroach. In another study conducted in a population of elderly urban patients with asthma, the presence of cockroach-specific serum IgE was associated with more severe asthma, as reflected by an increase in airway obstruction and hyperinflation [75]. In a Polish study it was observed that the concentration of Bla g 2 in houses was higher than previously reported in other European countries, and children with cockroach hypersensitivity had more often severe asthma than children with other allergies [76]. Previously identified allergens from Blatella germanica and Periplaneta americana (the most important domestic species), include Bla g 2 (inactive aspartic protease), Bla g 4 (calycin), Bla g 5 (glutathione-S-transferase), Bla g 6 (troponin), the Group 1 cross-reactive allergens Bla g 1 and Per a 1, Per a 3 (arylphorin), and Per a 7 (tropomyosin). On the basis of this finding, we suggested that community-based asthma intervention strategies should prioritize reducing cockroach allergen exposure.

## Sensitization to food allergens and severe asthma

Workers handling food products and derivatives are at increased risk of developing occupational asthma. Exposure to food allergens occurs primarily through inhalation of dust, steam, vapors and aerosolized proteins generated during the processing of foods. Most of the inhaled food allergies are IgE mediated, but objective evidence of asthma by monitoring peak expiratory flows during and off work or specific inhalation challenges usually provide a reliable diagnostic value [77]. Wheat may induce the well known baker’s asthma: albumins and globulins ( LTP, Tri a 14, alfa amylase inhibitor) are more involved than gluten (low – molecular – weight - glutenins, alfa, gamma omega 5 gliadin) [78]. Heat proteolytic resistant food allergens such as seafood (fish and shellfish), plants (LTP), spices, milk (casein), eggs (lysozyme, ovomucoid) and mushrooms can cause severe asthma attacks through inhalation [78, 79].

## Conclusions

Severe asthma remains a major health concern, due to its relevant social costs. Despite these critical aspects, there are still many unmeet needs with regard in particular the interpretation and comparison of the various studies carried out; for example, the different definitions of "severe asthma" adopted in the most important studies published so far (ENFUMOSA , SARP, TENOR, etc.) [3–8]. Different clinical and biological phenotypes of asthma are currently identified. Allergic asthma, with the atopic sensitization and the TH2 driven inflammation is the most frequent and the better known form of asthma [80]. Among the allergens that may cause asthma, some results to be more frequently associated to severe forms: molds, cockroach, pet dander, inhaled food-derived allergens. Also, “thunderstorm asthma” is frequently associated with severe symptoms due to a massive and sudden exposure to inhalant allergens. Thus, a detailed knowledge about the relevant allergens and causative factors is essential to properly diagnose, prevent, and treat the most severe forms of asthma.

There is evidence from some randomized controlled studies that ABPA treatment with systemic antifungal therapy can offer a therapeutic benefit to about 60% of patients [81].

Allergen immunotherapy (AIT) represents a valuable therapeutic option. A study of sublingual immunotherapy to *Dermatophagoides* in patients with allergic rhinitis with or without mild intermittent asthma showed an improved bronchial threshold to allergen challenge [29]. Another recent double-blind, randomized, placebo-controlled trial, conducted in 109 European trial sites and including 834 adults with HDM allergy–related asthma not well controlled by ICS or combination products, and with HDM allergy–related rhinitis, demonstrated that the addition of HDM SLIT tablets increased the time to first exacerbation during ICS reduction, with an estimated absolute reduction at 6 months of 9 to 10 percentage points; the reduction was primarily due to an effect on moderate exacerbations [30]. Further studies of these approaches are required in severe asthma. An alternative and clinically proven approach for atopic severe asthma is the use of the monoclonal antibody to IgE (omalizumab), which reduces circulating IgE and leads to down-regulation of its high affinity receptor FcεR1 on mast cells and basophils [31]. Different promising therapeutic options are also currently in development and undergoing clinical trials for the treatment of severe asthma, including anti-interleukin agents (mepolizumab, benralizumab, reslizumab, dupilumab, brodalumab, lebrikizumab) [32].
